# The Uses and Misuses of Mendelian Randomization in Clinical and Translational Science

**DOI:** 10.1016/j.jacbts.2024.06.005

**Published:** 2024-07-22

**Authors:** Paul C. Lee, Douglas L. Mann, Nathan O. Stitziel

**Affiliations:** a Division of Cardiology, Department of Medicine, Washington University School of Medicine, St. Louis, Missouri, USA; b Medical Scientist Training Program, Washington University School of Medicine, St. Louis, Missouri, USA; c Department of Genetics, Washington University School of Medicine, St. Louis, Missouri, USA

**Figure:**
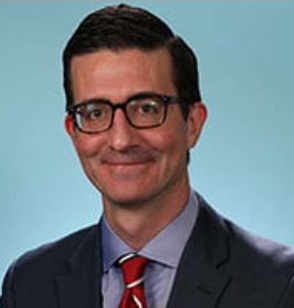

“Correlation does not imply causation” is a foundational axiom in the biomedical research enterprise that has important implications for translational science. Although it is obvious that therapeutically manipulating noncausal biomarkers is unlikely to alter disease risk, determining which relationships are causal can be difficult to assess. To this end, the randomized controlled trial (RCT) framework has been developed as the gold standard for determining if a correlative association between an exposure (eg, plasma low-density lipoprotein [LDL] cholesterol) and an outcome (eg, myocardial infarction) is due to a causal relationship. However, these trials are increasingly expensive and time-consuming. Accordingly, alternative methods to estimate causality have long been desired.

Over the past decade, a statistical technique has been adapted from the field of econometrics that uses human genetic variation as an instrumental variable (IV) in estimating the causal effects between exposures and outcomes.^1^^,^^2^ The attractiveness and power of this method, termed Mendelian randomization (MR), is in its direct analogy to that of the RCT framework. Stated simply, the MR approach asks if genetic variants that associate with altered levels of an exposure—which might be a biomarker like LDL cholesterol, a lifestyle factor like smoking, or a physiological trait like blood pressure—also associate with altered risk of disease. Because the genetic variants are randomly assorted at conception, MR (when appropriately used) circumvents issues that plague observational epidemiology studies, such as confounding and reverse causation. Thus, by using these genetic instrumental variables as proxies for modifiable risk factors, MR represents a powerful and elegant method to extract insights about potential causal relationships from large observational studies.

Fueled in part by increasing numbers of well-powered genome-wide association studies for both exposures and outcomes, over the past decade there has been an explosion in the use of MR to estimate causality in studies ranging from public health to basic science (Figure 1A). As examples, MR was used to estimate the causal effect of alcohol intake on risk of cardiovascular disease and to conclude that high-density lipoprotein cholesterol was not in a causal pathway for myocardial infarction.^3^^,^^4^ Drug-target MR studies have also been used to predict the therapeutic effects and side effects of PCSK9 inhibition, among others.^5^ The increased interest in and application of MR have also been driven by the development of tools that greatly facilitate MR analyses.

**Figure 1:**
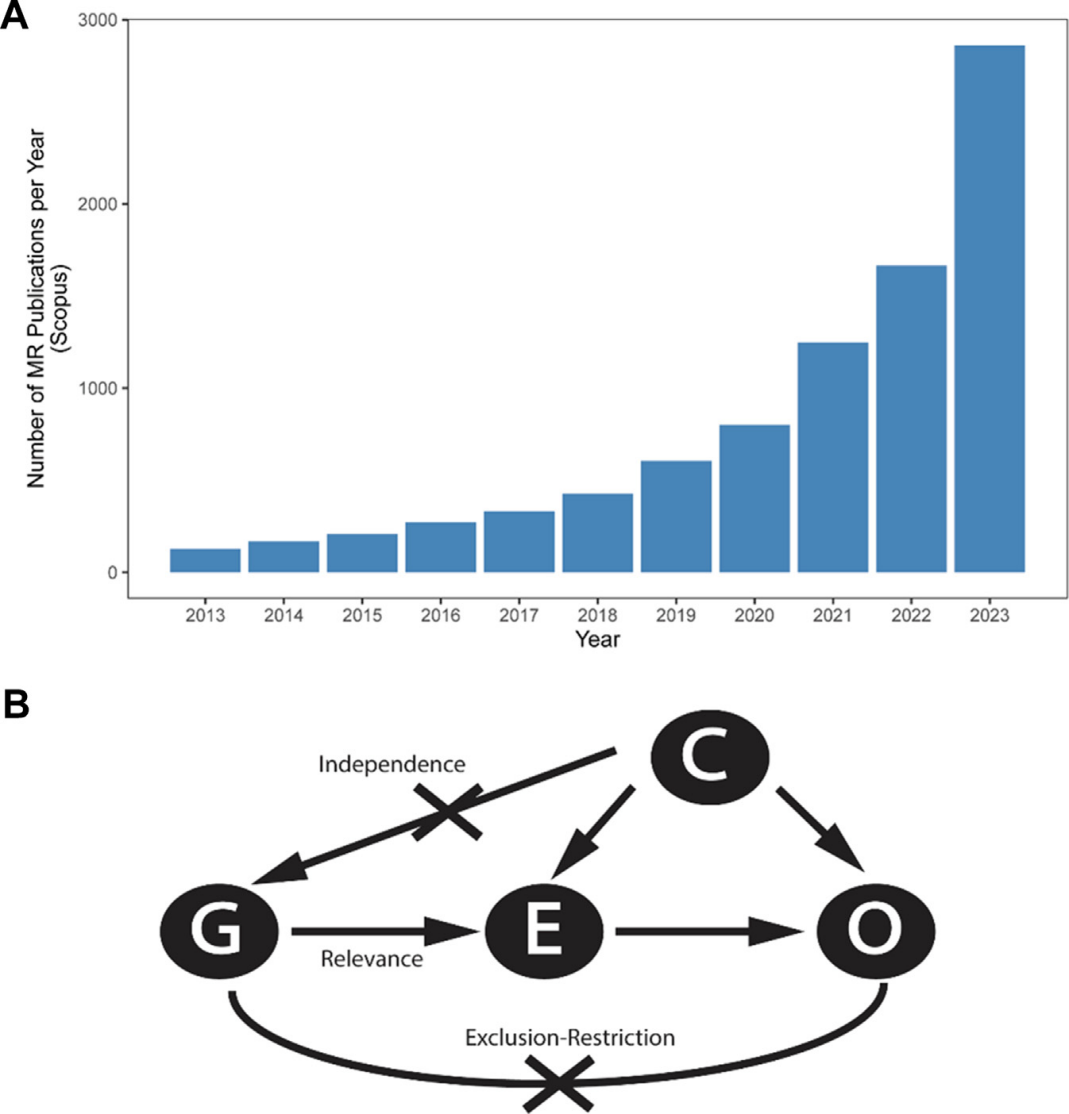
Overview of MR (A) Number of annual publications with “Mendelian randomization” as a key word in Scopus (www.scopus.com). (B) Directed acyclic graph illustrating the relationships between variables and the 3 core assumptions in Mendelian randomization (MR) analysis. C = Confounder trait; E = Exposure trait; G = Genetic variants; O = Outcome trait.

Despite its appeal, MR (like any technique in basic and translational research) relies on certain assumptions and can produce inaccurate or misleading conclusions about causality when those assumptions are violated. Here, we highlight some of the common pitfalls while performing, interpreting, and reporting MR analyses.

## Central MR Assumptions and Their Violations

The validity of MR relies on 3 core assumptions about the genetic variants selected as IVs^6^: 1) must be strongly associated with the exposure trait (the relevance assumption); 2) must not demonstrate any confounding association that could affect both the variant and the outcome trait (the independence assumption); and 3) must not associate with any additional traits that could plausibly affect the outcome trait (the exclusion-restriction assumption). It is critical that all 3 of these assumptions be given careful consideration to establish the reliability of the underlying MR model. The results of MR studies that violate any of the 3 assumptions should be interpreted cautiously.

First, the relevance assumption presumes that the genetic instrument is strongly associated with the exposure (Figure 1B) to avoid an effect known as weak-instrument bias. In practice, weak-instrument bias can be avoided by selecting only genetic variants associated with an exposure at a level of genome-wide significance (*P* < 5 × 10^−8^). Practically speaking, this requires large well-powered genetic association studies for exposures that often do not exist. As a result, some MR studies construct instruments using genetic variants that are more weakly associated with an exposure trait (eg, *P* < 5 × 10^−4^ or even *P* < 0.05). Depending on the overall MR study design, these weak instruments can either bias toward a confounded association or bias toward the null. Comprehensive MR study reports will include an estimate of the instrument’s strength known as the *F*-statistic. Generally, *F*-statistics >10 suggest low risk of weak-instrument bias (genome-wide significant variants used as IVs will have *F*-statistics near 30).

Second, the independence assumption presumes that there is not a confounding association that affects both the genetic instrument and the outcome trait (Figure 1B). Because the central tenet of MR relies on the random allocation of genetic variants during meiosis, this assumption is generally more difficult to violate. However, some possible violations include population stratification (ie, differences in the carrier frequency of genetic variants between subgroups within a larger population, typically due to ancestral differences) and assortative or nonrandom mating. Although the former can be guarded against by attempting to match the ancestries of participants in studies of exposures and outcomes, the latter can be difficult to detect and correct for in practice.

Third, the exclusion-restriction assumption presumes that the genetic instrument only influences the outcome through the exposure trait and not by other pathways (Figure 1B). This assumption covers the possibility of pleiotropic interactions in which a genetic variant may directly (or indirectly through confounders) affect the outcome. For example, a genetic instrument designed to test for a causal association between body mass index (BMI) and coronary artery disease (CAD) that is composed of variants that associate with both BMI and another cause of CAD (eg, LDL cholesterol) would violate the exclusion-restriction assumption, affecting the interpretation of the results. In reality, fully proving that the exclusion-restriction assumption has not been violated is very difficult because of our incomplete understanding of how genetic variants affect human traits. In practice, violating this assumption can be mitigated by selecting variants with strong a priori evidence of an exclusive effect on the exposure trait. Examples include using functional coding variants or only selecting variants within a given genetic locus for causal tests of specific genes or their protein products. Secondary analyses can also help detect whether the exclusion-restriction assumption has been violated. For instruments composed of multiple variants, heterogeneity of causal estimates should be estimated by calculating the Cochran Q or the *I*^2^ metric. Positive heterogeneity tests could indicate that pleiotropic variants have been included in the study. Fortunately, as interest in MR has increased, new methods such as MR-EGGER, MR-PRESSO, and others have been developed to detect and account for pleiotropy.

## Biological Plausibility and the Interpretation of Causal Relationships

With increasing access to approachable methods and data sets, MR can now be widely applied to test for causal relationships even in situations lacking any realistic biological plausibility. For example, genetic association studies have now been performed for thousands of human traits, theoretically allowing for millions of pairwise exposure-outcome MR analyses. To avoid testing ill-posed hypotheses, a good starting rationale for MR is the existence of a strong observational association. In that context, MR could be considered an extension analysis to estimate the presence of a causal association underlying the observational correlation. Similarly, MR could be used to assess the causal relationship between 2 traits that are connected by an established biological pathway. On the other hand, MR studies of traits with low heritability, traits with highly complex etiology, or a set of traits that lack underlying biological and/or epidemiological evidence connecting them should be interpreted cautiously, particularly if there is evidence that the core MR assumptions have been relaxed.

Related to biological plausibility, a common omission in the discussion of MR results is the lack of consideration for vertical pleiotropy as a possible explanation for the MR finding. Vertical pleiotropy is the presence of an association between the genetic variant and another exposure that is within the same exposure-outcome pathway. For example, a genetic instrument reflecting levels of an inactive enzymatic precursor may be causally associated with a disease in MR but that association may have been induced by the genetic instrument simultaneously reflecting levels of the active enzyme. Thus, although the presence of vertical pleiotropy does not violate the core IV assumptions, it can be an important consideration when interpreting the results.

## Avoiding Misuse in MR

As MR matures as a technique and becomes more widely used, efforts have been made to develop guiderails for analyzing and reporting MR results, and readers are recommended to consult them at the early stages of formulating an MR study. In particular, MR guidelines have recently been published that represent a comprehensive collection of best practices with plans for regular updates based on community feedback.^7^ Similarly, inspired by the EQUATOR (Enhancing the QUAlity and Transparency Of health Research) and STROBE (Strengthening the Reporting of Observational Studies in Epidemiology) guidelines, STROBE-MR has been developed as a checklist of items aimed to encourage more comprehensive and uniform reporting of MR results.^8^ The use of STROBE-MR has now been endorsed by journals such as *BMC Medicine* to accompany every MR study.^9^ At *JACC: Basic to Translation Science*, we similarly endorse the STROBE-MR guidelines when reviewing MR papers.

Even with these excellent safeguards in place, it is important to reiterate that the causal effect estimate obtained through MR should be interpreted with caution regardless of the precautions that were used. Although continual advancements in MR methods have been encouraging, from a practical standpoint it will never be possible to ensure that all core MR assumptions have been met, even when best practices have been followed. In this context, we believe that although MR is an efficient way to generate testable hypotheses about relationships between biological variables, it should not be used as a standalone substitute for a well-designed clinical trial or experiment.
